# Caspase-9 driven murine model of selective cell apoptosis and efferocytosis

**DOI:** 10.1038/s41419-023-05594-6

**Published:** 2023-01-24

**Authors:** Lena Batoon, Amy J. Koh, Rahasudha Kannan, Laurie K. McCauley, Hernan Roca

**Affiliations:** 1 Department of Periodontics and Oral Medicine, University of Michigan, School of Dentistry, Ann Arbor, MI USA; 2 Department of Pathology, University of Michigan, Medical School, Ann Arbor, MI USA

**Keywords:** Apoptosis, Bone

## Abstract

Apoptosis and efficient efferocytosis are integral to growth, development, and homeostasis. The heterogeneity of these mechanisms in different cells across distinct tissues renders it difficult to develop broadly applicable in vivo technologies. Here, we introduced a novel inducible caspase-9 (iCasp9) mouse model which allowed targeted cell apoptosis and further facilitated investigation of concomitant efferocytosis. We generated iCasp9^+/+^ mice with conditional expression of chemically inducible caspase-9 protein that is triggered in the presence of Cre recombinase. In vitro, bone marrow cells from iCasp9^+/+^ mice showed expression of the iCasp9 protein when transduced with Cre-expressing adenovirus. Treatment of these cells with the chemical dimerizer (AP20187/AP) resulted in iCasp9 processing and cleaved caspase-3 upregulation, indicating successful apoptosis induction. The in vivo functionality and versatility of this model was demonstrated by crossing iCasp9^+/+^ mice with CD19-Cre and Osteocalcin (OCN)-Cre mice to target CD19^+^ B cells or OCN^+^ bone-lining osteoblasts. Immunofluorescence and/or immunohistochemical staining in combination with histomorphometric analysis of EGFP, CD19/OCN, and cleaved caspase-3 expression demonstrated that a single dose of AP effectively induced apoptosis in CD19^+^ B cells or OCN^+^ osteoblasts. Examination of the known efferocytes in the target tissues showed that CD19^+^ cell apoptosis was associated with infiltration of dendritic cells into splenic B cell follicles. In the bone, where efferocytosis remains under-explored, the use of iCasp9 provided direct in vivo evidence that macrophages are important mediators of apoptotic osteoblast clearance. Collectively, this study presented the first mouse model of iCasp9 which achieved selective apoptosis, allowing examination of subsequent efferocytosis. Given its unique feature of being controlled by any Cre-expressing mouse lines, the potential applications of this model are extensive and will bring forth more insights into the diversity of mechanisms and cellular effects induced by apoptosis including the physiologically important efferocytic process that follows.

## Introduction

Apoptosis and efficient clearance are integral to tissue development, homeostasis and repair [1, 2]. Aberrations in efferocytosis underlie a variety of chronic diseases and cancers [3, 4]. Although billions of cells are routinely undergoing apoptosis, they are hardly observable in tissues because efferocytosis is a rapid and highly efficient process. This process is largely performed by cells with high phagocytic capacity (macrophages and dendritic cells) [1], but other phagocytes including neutrophils [5] are also capable efferocytes. In bone, efferocytosis remains under-explored, but both bone-resorbing osteoclasts [6] and macrophages [7–9] have been implicated mostly via utilization of in vitro techniques.

An incredible amount of knowledge regarding the key signaling pathways that regulate apoptosis and subsequent efferocytosis has been gained within the past century. This knowledge advancement recognized that cell clearance mechanisms are highly heterogeneous [2]. Different cell types undergo apoptosis at different rates and likely have different sets of “find-me” cues. Moreover, there is great variation in the types of professional, non-professional and specialized phagocytes that reside in different tissues [1] which likely contribute to the efferocytic process at varying levels. Due to this heterogeneity, a key challenge in studying efferocytosis is the difficulty in developing strategies for tracking and targeting this process in vivo [2].

This study aimed to generate a broadly applicable mouse model where target cells can be selectively induced to undergo apoptosis in order to investigate efferocytosis using the initiator enzyme for intrinsic apoptosis, caspase-9, that is active in dimeric form [10]. The model generated here utilized the Cre/lox strategy whereby the inducible caspase-9 (iCasp9) is expressed by the target Cre-expressing cells and can be induced via a dimerizing compound. Using this model, we identified and described experimental strategies that achieved effective cell apoptosis in both soft and hard tissues, which further facilitated investigation of concomitant efferocytosis.

## Materials And methods

### iCasp9 model design and construction

**Fig. 1:**
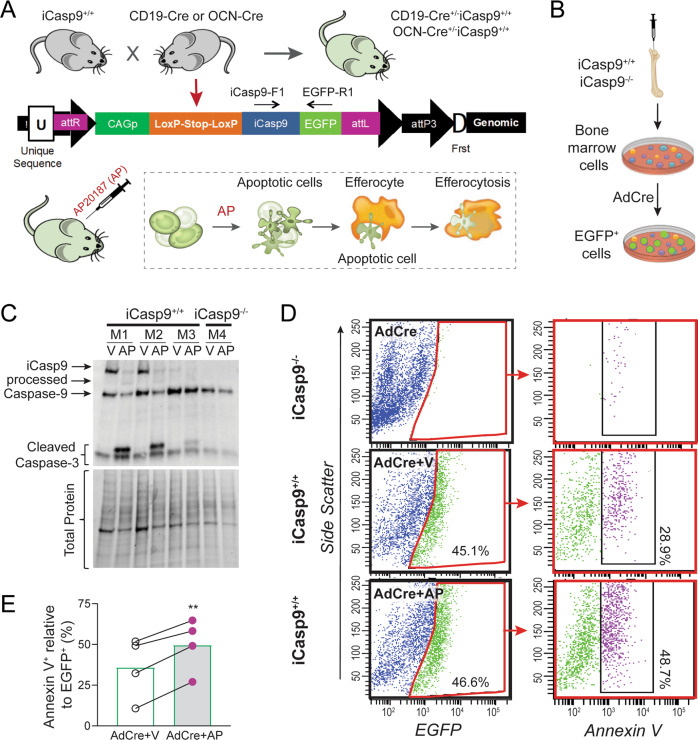
iCasp9 mouse model and in vitro validation. **A** iCasp9^+/+^ mice crossed with Cre mouse lines (CD19-Cre or OCN-Cre) recombines the LoxP sites causing excision of the stop codon in the targeted construct allowing expression of iCasp9 and EGFP in Cre-expressing cells. The idea of the model is to inject AP20187 (AP) to dimerize and activate iCasp9, inducing apoptosis of target cells and subsequent efferocytosis. **B** Bone marrow cells from iCasp9^+/+^ mice and iCasp9^−/−^ littermates were cultured and transduced with adenovirus-Cre (AdCre) for 48 h. Isolated proteins were analyzed by **C** Western blot analysis showing expression of iCasp9, processed iCasp9, endogenous caspase-9 and cleaved caspase-3. Individual mice are indicated (M1–M4). (**D** Representative flow cytometry plots and proportion of EGFP^+^ (left plots) and Annexin V^+^ cells (right plots). (**E**) Percentage of Annexin V^+^ signals in EGFP-expressing cells. Statistical significance was determined using paired *t*-tests comparing AP- and vehicle-treated cultures from the same animal. **p < 0.01. *n* = 4 mice/group.

A proprietary TARGATT™ knock-in technology (Applied StemCell Inc., Milpitas, CA, USA) was used to create a transgenic mouse model expressing iCasp9. Cre-inducible expression of iCasp9 encoded in the CAGpromoter-Lox-STOP-Lox-iCasp9-T2A-EGFP cassette was made by cloning a synthetic fragment containing an F36V/F36V (binding domain of AP20187/AP) fused with caspase-9 cDNA [11] and followed by T2A-EGFP into a vector containing the Cre-targeted recombination sequence Lox-STOP-Lox (Fig. 1A, S1). This construct was integrated into the mouse Rosa26 locus. Detailed procedure of iCasp9 mouse generation is provided in Supplementary Material.

### Experimental animals

Animal experiments were approved by the University of Michigan (UM) Institute for Animal Care and Use Committee following the NIH Guide for the Care and Use of Laboratory Animals. Animals were provided free access to food and water and housed in 12 h light/dark cycle. Homozygous iCasp9^+/+^ mice were bred with CD19-Cre (B6.129P2(C)-*Cd19*^*tm1(cre)Cgn*^/J) or Osteocalcin (OCN)-Cre (B6.FVB-Tg(BGLAP-cre)1Clem/J) mice obtained from Jackson Laboratory (Bar Harbor, ME, USA). Male and female mice at three-four weeks were used as no gender differences were detected in the tissues examined at this age. Custom-generated primers (Life Technologies, Eugene, OR, USA) were used to confirm Cre presence (Supplemental Table 1). C57Bl/6J mice were maintained in-house.

### Adenoviral transduction and Western blot

Bone marrow (BM) cells from iCasp9^+/+^ and iCasp9^−/−^ mice were cultured as previously described [7] and transduced with Cre-expressing adenovirus (AdCre, 400MOI; UM Vector Core, Ann Arbor, MI, USA). After 48 h, cells were washed with αMEM containing 10% fetal bovine serum (FBS) and 1% penicillin/streptomycin/glutamine twice. Following 48 h incubation, cells were treated with AP (10 µM)/vehicle overnight. Protein collection and Western blot analyses were performed as previously described [12].

### In vivo AP administration and tissue collection

AP (Takara Bio, San Jose, CA, USA) dosing solutions (1.25 mg/ml or 2.5 mg/ml) were prepared as per manufacturer’s recommendations. Similar composition without AP was used as “vehicle”. Mice were randomly allocated to “AP” or “vehicle” groups. AP/vehicle was administered intraperitoneally at 20 µl/g body weight to achieve a dose of 25 µg/g from 1.25 mg/ml AP solution or 50 µg/g from 2.5 mg/ml solution. Mice were sacrificed at 9 or 24 h post injection. Spleens and lumbar vertebrae were fixed in 4% paraformaldehyde. Blood was collected via cardiac puncture. BM cells were collected with FACS buffer (phosphate-buffered saline, 1 mM Ethylenediaminetetraacetic acid, 2% FBS).

### Cell culture and flow cytometry

OCN-Cre^+/−^iCasp9^+/+^ and OCN-Cre^−/−^ mouse calvarial cells were collected [13] and incubated with vehicle or AP (20 µM) for 1 h. Cells were washed [7] and added to BM macrophage (BMM) cultures propagated as previously described [7]. Cells were collected and stained with anti-F4/80-APC antibody (Abcam, Cambridge, MA, USA) for 30 min. Cells were washed and analyzed using FACSAria (BD Biosciences, Franklin Lakes, NJ, USA).

Blood cell suspensions were prepared [14], stained using the Annexin V/PI staining kit as per manufacturer’s instructions (BD, San Diego, Pharmingen, CA, USA) and analyzed via flow cytometry. Non-Cre (EGFP^-^) cells and isotype controls established the gates for analysis.

### Immunofluorescence, immunohistochemistry and histological staining

Tissues were paraffin-embedded [15]. Spleen or decalcified bone sections (5 µm) were collected. Primary antibody information including catalog number, dilution, incubation period and antigen retrieval methods are listed in Supplemental Table 2. Non-specific binding was blocked using 10% normal goat serum and 10% FBS in Tris-buffered saline (TBS) for 1 h. For immunofluorescence staining, the species-matched secondary antibody used were goat anti-chicken-AlexaFluor488, goat anti-rat-AlexaFluor647, goat anti-rat-Cy3 or goat anti-rabbit-AlexaFluor680 from Thermo Fisher Scientific, Waltham, MA, USA. Slides were mounted using Prolong Gold mounting medium with DAPI (Thermo Fisher Scientific). Fluorescent [16] and colorimetric TRAP [17] staining were performed as previously described. Hematoxylin and Eosin (H&E) staining was performed using routine protocol. For immunohistochemical detection of EGFP and cleaved caspase-3, endogenous peroxidase activity was blocked using 3% hydrogen peroxide/TBS. After primary antibody, species-matched biotinylated secondary antibody was added followed by HRP-conjugated streptavidin (Vectorlabs, Burlingame, CA, USA) for 30 min each. Sections were incubated with DAB substrate (Abcam) and counterstained with Mayer’s hematoxylin (Sigma-Aldrich, St. Louis, MO, USA). Slides were imaged using the Leica THUNDER imaging system (Leica Microsystem, ZQQ, DE).

### Histomorphometry

EGFP, CD19, F4/80 and CD11c staining in whole spleen sections were quantified with ImageJ using 10X tiled images. For CD11c staining within B cell follicles, regions of interest (ROI) were set using images from the EGFP channel. The appropriate intensity threshold was identified using a vehicle control section and applied across all samples. The “area fraction” (percentage of pixels in the ROI that fall within the selected threshold) was recorded. Cleaved caspase-3^+^ signals in the spleen were analyzed with AIVIA (Leica Microsystem) using 20X tiled images of the entire section. The pixel-based iterative training process within the software was used to develop an algorithm that detected DAB staining (brown) from hematoxylin (nuclei/blue) and white spaces (extracellular matrix, blood vessels and adipocyte ghosts). The trained algorithm detected staining with high precision (Fig. S3A, B) and was batch-applied across all samples. Resulting values were converted to number per mm^2^ of tissue.

Analyses of OCNCre^+/−^iCasp9^+/+^ sections were performed blindly in ImageJ using 20X tiled images of the vertebrae. The percent length of EGFP^+^ surface per trabecular bone surface (Tb.BS), OCN^+^ surface per Tb.BS, TRAP^+^ osteoclast surface per Tb.BS, F4/80^+^ surface per Tb.BS, the number of F4/80^+^EGFP^+^ or Ly6G^+^ cells per Tb.BS and number of cleaved caspase-3^+^ signal per vertebral area were manually quantified. Assessment of F4/80^+^ cells containing intracellular EGFP^+^ signal was supported by z-stack imaging. Measurements were taken in the entire region between the two cartilaginous endplates. EGFP^+^ staining in BM stromal cells (BMSC) and within the bone (osteocytes) were enumerated and converted to number per mm^2^ of BM or bone, respectively. In both superior and inferior cartilaginous endplates, EGFP^+^ staining was enumerated and expressed as number per mm^2^ of tissue.

### Statistical analyses

Statistically significant differences were determined using paired t-test or two-way analysis of variance (ANOVA) with Sidak’s multiple comparison post-test where individual variances are accounted for in each comparison using PRISM 9 (GraphPad Software, Inc., La Jolla, CA, USA). Parametric tests were used for datasets that passed the Shapiro-Wilk normality test. When sample size was too small for normality test, normal distribution was assumed. A value of *p* < 0.05 was considered statistically significant. Group sizes are indicated in figure legends. Graphs display mean and standard deviation, and each data point represents an independent mouse.

## Results

### iCasp9 mouse model and in vitro validation

iCasp9 construct functionality was verified in vitro using iCasp9^+/+^ or iCasp9^−/−^ mouse BM cells (Fig. 1B). Western blot analysis confirmed expression of the iCasp9 transgene when iCasp9^+/+^ BM cells were transduced with AdCre. Further treatment of these cells with AP resulted in processing of iCasp9 and upregulation of cleaved caspase-3 (Fig. 1C, Fig. S2), a commonly used marker of apoptotic cells where caspase-3 is activated via cleavage by caspase-9 [18]. Cells isolated from iCasp9^−/−^ mice did not show expression of iCasp9 or cleaved caspase-3 (Fig. 1C, Fig. S2). Flow cytometry of cells from similar samples demonstrated EGFP expression in iCasp9^+/+^ mouse BM cells transduced with AdCre (Fig. 1D) and AP treatment increased the percentage of dead Annexin V^+^ cells indicating increased apoptosis (Fig. 1D, E).

### A single AP injection in CD19-Cre^+/−^iCasp9^+/+^ mice induced CD19^+^ cell apoptosis

**Fig. 2:**
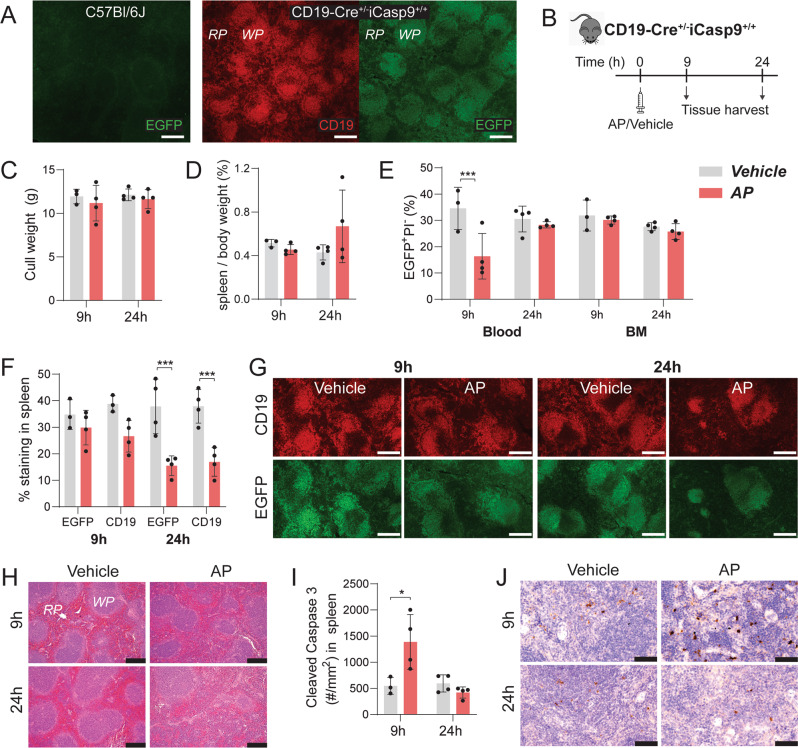
AP treatment in CD19-Cre^+/−^iCasp9^+/+^ mice induced CD19^+^ cell apoptosis in the spleen. **A** Immunofluorescent (IF) images of serial spleen sections from untreated CD19-Cre^+/−^iCasp9^+/+^ mice showing CD19 and EGFP staining with aged-matched C57Bl/6J mouse spleen as a negative control for EGFP expression. RP—red pulp, WP—white pulp. **B** Schematic detailing the experimental design including timing of AP/vehicle injection and tissue harvest. **C** Body weight and **D** percent spleen per body weight at the experimental endpoint. **E** Percentage of live (PI^−^) EGFP^+^ cells in blood and BM at 9 and 24 h post vehicle or AP treatment as analyzed by flow cytometry. **F** Quantification of EGFP^+^ and CD19^+^ staining and **G** representative IF images of serial spleen sections. **H** H&E images of spleen sections with the red pulp (RP) and white pulp (WP) indicated. **I** Quantification of cleaved caspase-3^+^ staining in the entire spleen section expressed as number per tissue area, and **J** representative IHC images. Statistical significance was determined using two-way ANOVA with Sidak’s multiple comparisons test. *p < 0.05; ***p < 0.001. Error bars represent standard deviation. *n* = 3–4 mice/group. Scale bar: 200 µm (**A**, **G**, **H**), 50 µm (**J**).

In vivo functionality of iCasp9^+/+^ mice was examined in CD19Cre^+/−^iCasp9^+/+^ mice to target CD19^+^ B cells [19]. The distribution of cells that underwent Cre-mediated recombination was examined via EGFP expression. As expected, no EGFP expression was detected in Cre-negative C57Bl/6J spleen (Fig. 2A). In CD19Cre^+/−^iCasp9^+/+^ mouse spleen, CD19 and EGFP exhibited an overlapping expression pattern predominantly within the white pulp, confirming successful recombination in the target cells. Mice were treated once with vehicle or AP (25 µg/g) and tissues were harvested at 9 or 24 h post injection (Fig. 2B). Despite being 2.5× higher than the dose recommended by the manufacturer, this AP dose had no impact on body (Fig. 2C) or spleen (Fig. 2D) weight. Flow cytometry analysis showed AP administration reduced circulating EGFP^+^ cells at 9 h post treatment but their frequency returned to baseline levels after 24 h (Fig. 2E). In BM, no difference in EGFP^+^ cells was detected between the groups at either time-point. Immunofluorescence staining of the spleen revealed a significant reduction in both EGFP and CD19 staining at 24 h post AP injection (Fig. 2F, G). This reduction was associated with disruption of the splenic architecture where red and white pulps were no longer discernible (Fig. 2H).

**Fig. 3:**
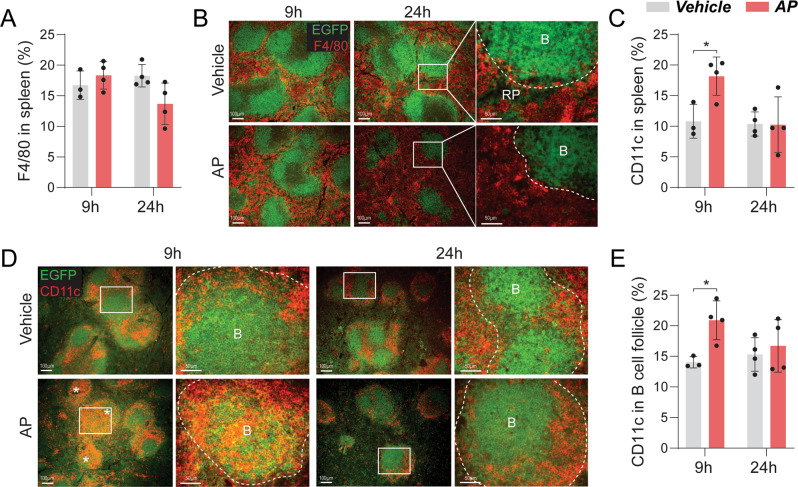
Induced CD19^+^ cell apoptosis was associated with infiltration of CD11c^+^ cell into splenic B cell follicles. **A** Quantification and **B** representative images of F4/80^+^ staining in whole spleen sections from CD19-Cre^+/−^iCasp9^+/+^ mice at 9 or 24 h post vehicle or AP treatment. RP—red pulp, B—B cell follicle. **C** Quantification and **D** representative images of CD11c^+^ staining in spleen including magnified images of B cell follicles. *denotes B cell follicles with CD11c^+^ cell infiltration. **E** Quantification of CD11c^+^ staining localized within B cell follicles. Statistical significance was determined using two-way ANOVA with Sidak’s multiple comparisons test. *p < 0.05. Error bars represent standard deviation. *n* = 3–4 mice/group. Scale bar: 50 µm for magnified regions and 100 µm for other images.

A marked increase in cleaved caspase-3^+^ signals was detected at 9 h (Fig. 2I, J). Examination of common efferocytes revealed no significant change in F4/80^+^ macrophage frequency (Fig. 3A, B). CD11c expression (predominantly dendritic cells) was significantly increased in the spleen at 9 h but returned to baseline level at 24 h (Fig. 3C, D). Further assessment of CD11c^+^ cell distribution demonstrated infiltration into B cell follicles at 9 h (Fig. 3D, E). Lymph nodes were also examined but no significant differences were found (Fig. S3C, D).

### A single AP injection in OCN-Cre^+/−^iCasp9^+/+^ mice induced osteoblast-specific apoptosis

**Fig. 4:**
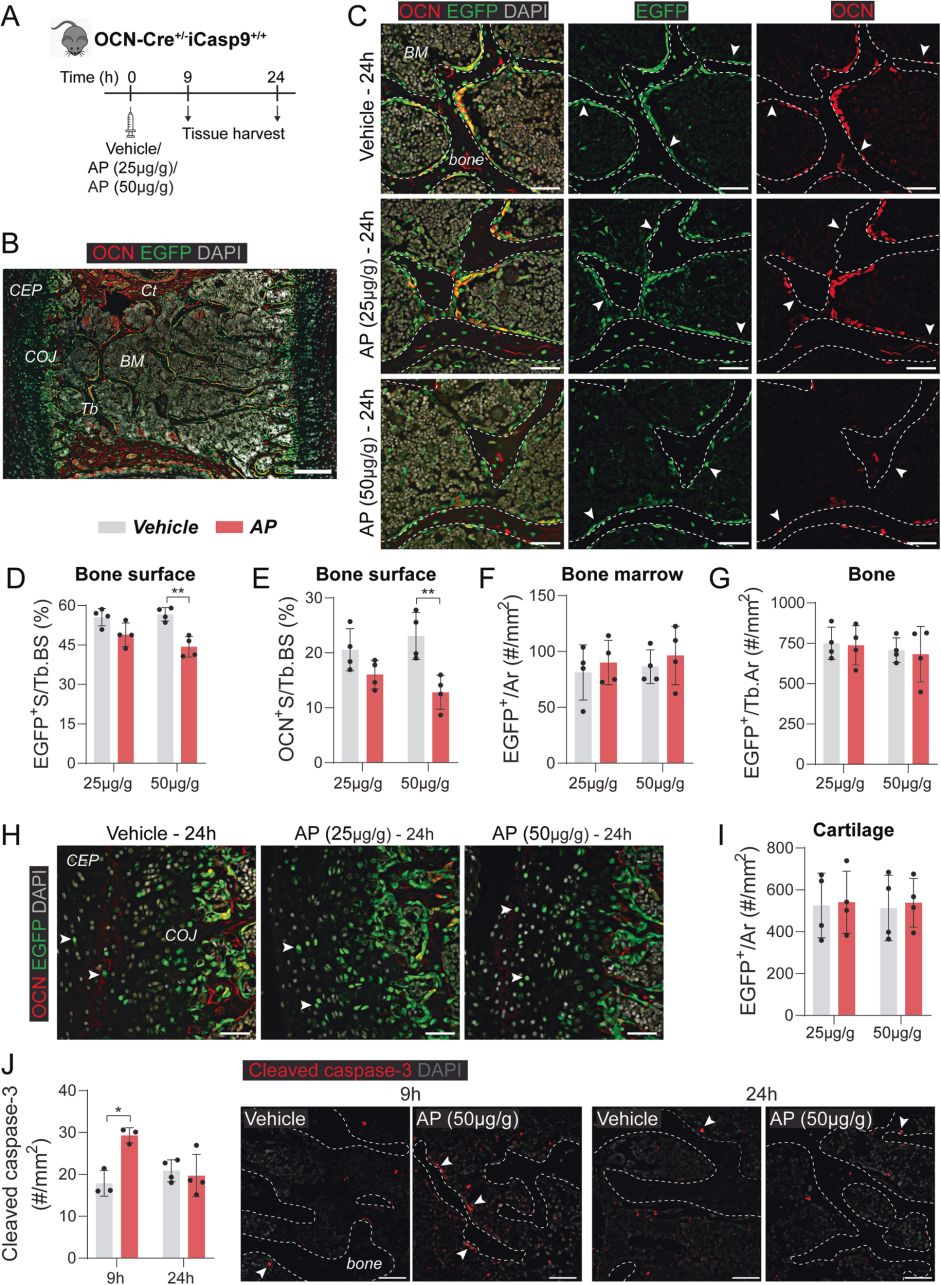
A single injection of AP in OCN-Cre^+/−^iCasp9^+/+^ mice resulted in selective osteoblast apoptosis. **A** Schematic detailing the experimental design including dose and timing of AP/vehicle injection and tissue harvest. **B** Immunofluorescent (IF) image of a lumbar vertebra from a vehicle-treated OCN-Cre^+/−^iCasp9^+/+^ mouse showing distribution of EGFP and OCN expression. CEP—cartilaginous endplate, COJ—chondro-osseous junction, Tb—trabecular bone, BM—bone marrow, Ct—cortical bone. **C** IF images showing EGFP and OCN expression in AP/vehicle-treated OCN-Cre^+/−^iCasp9^+/+^ mouse vertebrae at 24 h post injection. Dotted lines denote bone. Arrowheads indicate OCN^neg^EGFP^+^ bone lining cells. Quantification of **D** EGFP^+^ surface (S) per trabecular bone surface (Tb.BS), **E** OCN^+^ S per Tb.BS, **F** number of EGFP^+^ signal in the bone marrow (BM) per tissue area (Ar), and **G** EGFP^+^ osteocytes expressed as number per trabecular area (Tb.Ar) at 24 h post treatment. **H** Representative images and **I** quantification of the number of EGFP^+^ signals (arrowheads) within the cartilaginous endplates. **J** Quantification and representative images of cleaved caspase-3 staining at 9 and 24 h post treatment with AP (50 µg/g) or vehicle. Arrowheads indicate staining adjacent to bone surfaces. Statistical significance was determined using two-way ANOVA with Sidak’s multiple comparisons test. *p < 0.05; **p < 0.01. Error bars represent standard deviation. *n* = 3–4 mice/group. Scale bar: 200 µm (**B**), 50 µm (**C**, **H**, **J**).

The versatility of the model was assessed by targeting cells in another distinct tissue (bone). OCN-Cre mice have been widely used to study osteoblasts, but have also been shown to target some BMSCs [20]. A similar experimental strategy described in the previous section was first employed in OCNCre^+/−^iCasp9^+/+^ mice but no reportable differences between groups were noted with this regimen (Fig. 4A–G). Thus, a higher AP dose (50 µg/g) was also tested (Fig. 4A). Dual EGFP and OCN staining of the vertebra confirmed OCN^+^ bone-lining cells expressed EGFP (Fig. 4B–E). However, EGFP positivity also occurred in: OCN-negative bone-lining cells, some chondrocytes in the cartilage endplates and chondro-osseous junction, BMSCs, and some osteocytes (Fig. 4B, C). The impact of AP injection on these populations was determined separately. The frequencies of EGFP^+^ cells in the BM (Fig. 4F), EGFP^+^ osteocytes (Fig. 4G) and EGFP^+^ chondrocytes (Fig. 4I) remained unchanged even with 50 µg/g AP. Conversely, bone-lining EGFP^+^ cells were substantially reduced (Fig. 4D). EGFP^+^ cells covered approximately 50% of the bone surface (Fig. 4D) while OCN^+^ cells covered about 20% (Fig. 4E), indicating that OCN-expressing osteoblasts only constitute 40% of EGFP^+^ bone-lining cells. There was nearly 50% reduction in OCN^+^ cells (Fig. 4E) while the reduction in EGFP^+^ bone lining cells was only about 7-8% (Fig. 4D), suggesting that the decrease in EGFP^+^ cells was mainly due to loss of OCN^+^EGFP^+^ cells. These findings indicate that despite occurrence of Cre-mediated recombination in other cell types, mature OCN-expressing osteoblasts were selectively targeted following a single 50 µg/g dose of AP. Interestingly, when vertebrae were assessed at an earlier time-point (9 h), while no differences in EGFP^+^ cell frequency were detected (Fig. S4A–D), a significant increase in cleaved caspase-3^+^ apoptotic signals was observed, many of which were adjacent to the bone (Fig. 4J). Cleaved caspase-3^+^ staining was no longer different between the groups at 24 h.

### Efferocytosis of apoptotic osteoblasts in OCNCre^+/−^iCasp9^+/+^ mice was mediated by macrophages

**Fig. 5:**
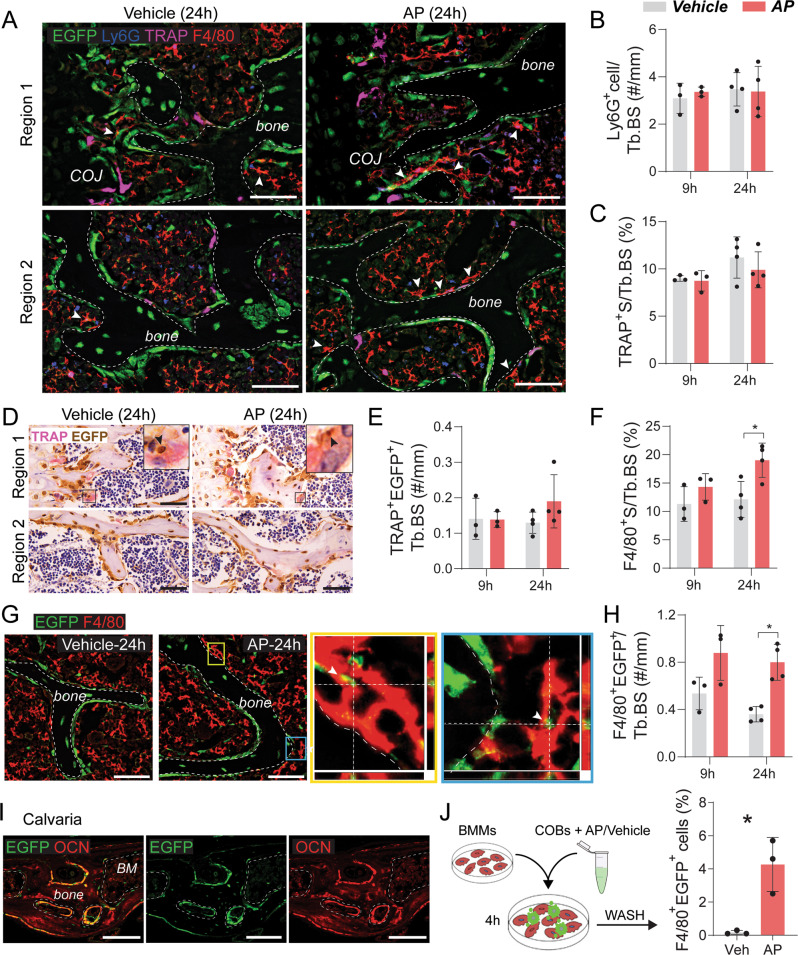
Macrophages mediate efferocytosis of apoptotic osteoblasts. **A** Lumbar vertebrae from vehicle- or AP (50 µg/g)-treated OCN-Cre^+/−^iCasp9^+/+^ mice showing multiplex immunofluorescent (IF) staining for EGFP (green), Ly6G (blue), TRAP (magenta) and F4/80 (red) expression. Arrowheads indicate F4/80^+^ macrophages directly associated with bone or EGFP^+^ osteoblasts. COJ—chondro-osseous junction. Quantification of **B** the number of Ly6G^+^ signals directly associated with trabecular bone or EGFP^+^ bone-lining cells using the multiplex IF images (**A**). **C** Quantification of TRAP^+^ surface (S) per trabecular bone surface (Tb.BS) using **D** colorimetric TRAP (pink) assay in combination with immunohistochemical detection of EGFP (brown). Arrowheads indicate EGFP^+^ signal within TRAP^+^ osteoclasts in the primary spongiosa (Region 1) that were quantified in (**E**). **F** Quantification of percent F4/80^+^ surface (S) per Tb.BS using the multiplex IF images (**A**). **G** Representative images of dual EGFP and F4/80 staining. Yellow- and blue-boxed panels are magnified z-stack images of regions in “AP” showing F4/80^+^ cells encapsulating EGFP^+^ remnants (arrowheads). **H** Number of F4/80^+^ cells containing EGFP^+^ remnants located on or within one cell from bone surfaces. **I** IF images showing expression of EGFP and OCN in OCN-Cre^+/−^iCasp9^+/+^ mouse calvaria. **J** Flow cytometry analysis of bone marrow-derived macrophages (BMMs) cultured for 4 h with calvarial osteoblasts (COBs) that were pre-treated with AP/vehicle. Statistical significance was determined using two-way ANOVA with Sidak’s multiple comparisons test or paired *t*-tests comparing AP-treated culture with vehicle-treated control from the same animal. *p < 0.05. Error bars represent standard deviation. *n* = 3–4 mice/group. Scale bar: 50 µm (**A**, **D**, **G**), 20 µm (**I**).

Given a single 50 µg/g dose of AP achieved selective osteoblast apoptosis (Fig. 4), this dose was utilized to identify the cells mediating efferocytosis of apoptotic osteoblasts. EGFP staining patterns in the cartilaginous endplate, chondro-osseous junction and primary spongiosa of AP- and vehicle-treated mouse vertebrae appeared comparable (Fig. S4E, Fig. 5A). In the middle region primarily consisting of trabeculae, EGFP staining in “AP” sections were visibly less than in the control group (Fig. S4E, Fig. 5A), consistent with a site where cells had undergone apoptosis. Large TRAP^+^ cells were mainly confined to the chondro-osseous junction and primary spongiosa where EGFP staining appeared unchanged (Fig. S4E, Fig. 5A). Unlike TRAP^+^ osteoclasts, Ly6G^+^ neutrophils and F4/80^+^ macrophages were abundant throughout the BM located in the cavities of trabecular bone (Fig. S4E, Fig. 5A). There was no difference in the distribution nor the number of Ly6G^+^ cells on bone surface between AP- and vehicle-treated groups at 9 or 24 h post treatment (Fig. 5A, B). Z-stack imaging of multiple regions across the vertebra failed to detect any Ly6G^+^ cells with intracellular EGFP remnant (data not shown).

To examine whether osteoclast frequency was altered, TRAP-covered bone surface was examined. The fluorescent precipitate from ELF97 substrate is restricted to TRAP^+^ granules [16] therefore, the classical colorimetric TRAP assay which has diffuse staining pattern was used for improved accuracy. There was no difference in TRAP^+^ surface per bone surface (Fig. 5C). Rare TRAP^+^ cells containing EGFP^+^ remnants were detected (Fig. 5D, E), but their frequency was not altered by AP treatment (Fig. 5E).

Quantification of F4/80^+^ macrophages located within one cell from the bone surface showed a significant increase at 24 h in AP-treated animals compared with the controls (Fig. 5F). Further assessment of these macrophages via z-stack imaging revealed that many contained EGFP^+^ remnants (Fig. 5G), consistent with macrophage efferocytosis. The frequency of these efferocytic F4/80^+^EGFP^+^ macrophages on or adjacent to bone was more than threefold higher than TRAP^+^EGFP^+^ cells (Fig. 5E) and was further increased with AP treatment (Fig. 5H). Macrophage efferocytosis of apoptotic osteoblasts was further demonstrated in vitro by culturing BMMs with AP- or vehicle-treated EGFP^+^ calvarial osteoblasts. The expression patterns of EGFP and OCN were almost completely overlapping in the calvaria (Fig. 5I) likely due to the absence of chondrocytes and fewer BM cells both of which have subsets that exhibited EGFP expression in the vertebrae (Fig. 4). Flow cytometry of BMMs cultured with vehicle-treated calvarial osteoblasts showed negligible frequency of F4/80^+^EGFP^+^ cells (≤0.3%, Fig. 5J) while co-culture with AP-treated calvarial osteoblasts resulted in elevated F4/80^+^EGFP^+^ cells, supporting increased efferocytosis.

## Discussion

A key challenge in efferocytosis research is the heterogeneity of the process across different tissues, rendering it difficult to develop widely applicable in vivo technologies. Here, we introduced a novel mouse model which allowed targeted cell apoptosis and facilitated detection of concomitant efferocytosis. We generated the iCasp9^+/+^ mice with conditional expression of chemically inducible caspase-9 protein that is triggered in the presence of Cre. The functionality and versatility of this model was demonstrated using CD19-Cre and OCN-Cre mice where a single dose of AP effectively induced B cell or osteoblast apoptosis and triggered dendritic cell infiltration or macrophage recruitment, respectively. Of particular significance, the use of iCasp9 mice provided direct in vivo evidence that macrophages are important mediators of osteoblast clearance in bone—a tissue where efferocytosis remains under-explored.

We exploited the function of caspase-9 as the initiator of intrinsic apoptosis [10]. The efficient induction of cell death via iCasp9 has been demonstrated in clinical settings and non-rodent animal models. iCasp9 is being actively scrutinized as a safety switch in T-cell therapies (NCT03373071) [21–23] and as a promising killing strategy in cancer management [24, 25]. Animal models of iCasp9 have been generated in frog [26] and chicken [27], but to our knowledge, the current study is the first to report utilization in mice. This model can be easily adapted by many given the extensive use of Cre mouse lines in biomedical research. Commonly used cell ablation mouse models include the herpes simplex virus thymidine kinase gene/ganciclovir approach [28] and transgenic expression of a primate diphtheria toxin (DT) receptor [29]. Both approaches induce a mixture of apoptotic and necrotic mechanisms [30, 31]. The iCasp9 model ensures selective activation of the caspase-9-mediated apoptotic pathway and utilizes a cell-permeable non-immunogenic compound (AP) and therefore, does not produce neutralizing antibodies as observed with DT administration [32].

Selective targeting of osteoblasts was achieved in OCN-Cre^+/−^iCasp9^+/+^ mice. OCN-Cre mice have been extensively used to examine osteoblasts but they also impact osteocytes, CXCL12-abundant reticular cells and pericytes [20]. We extended this characterization by showing that a proportion of chondrocytes in cartilage endplates are also targeted. Consequently, it was crucial to examine all populations in our model. The EGFP expression did not directly overlap with anti-OCN antibody staining as reported previously [20]. This could be related to differences in the regulation of endogenous OCN expression versus the transgenic control of EGFP which reflects both mRNA and protein—a phenomenon that occurs in transgenic reporters [33]. Interestingly, while various cell types expressed iCasp9 as reflected by EGFP, only bone-lining osteoblasts were reduced by AP. Examining the cause of cell-to-cell variability was outside the scope of this study, however, apoptosis heterogeneity in iCasp9 Hela cell line was reported to be contributed to by the initial iCasp9 expression level and ratio of Caspase-3 and its inhibitor XIAP [34].

A strength of our study was the examination of efferocytosis following effective apoptosis induction. The different AP doses utilized to achieve effective CD19^+^ and OCN^+^ cell death reiterates the variability of apoptosis in different cells/tissues and indicates that chemical inducer titration is required when iCasp9 mice are used to target other cell types. In both CD19-Cre^+/−^iCasp9^+/+^ and OCN-Cre^+/−^iCasp9^+/+^ models, increased cleaved caspase-3 was detected at 9 h. Although the kinetics of effector caspase activation are highly variable, our finding corroborates previously demonstrated timing of 2–12 h following a death stimulus [2]. The return of cleaved caspase-3 expression to baseline level at 24 h suggested that efferocytosis had occurred between the time points assessed. “Find-me” signals are released following executioner caspase activation [35, 36], allowing rapid efferocyte recruitment. Tissues are interspersed with networks of phagocytes/efferocytes and their positioning is likely an important factor for maximizing their interaction with dying cells and therefore clearance efficiency. In CD19-Cre^+/^iCasp9^+/+^ mice, F4/80^+^ macrophages in splenic red pulp [14] and CD11c^+^ dendritic cells largely positioned within the marginal zone [37] were examined. CD19^+^ B cell apoptosis was accompanied by a significant CD11c^+^ cell infiltration into B cell follicles. Dendritic cells are capable efferocytes within splenic white pulp [38]. While efferocytosis was not directly assessed in our sections given the difficulty to distinguish individual cells, the spatiotemporal nature of their infiltration following CD19^+^ cell apoptosis likely reflects an efferocytic response. CD169^+^F4/80^neg^ macrophages within the splenic marginal zone have known efferocytic function [39] but we were unable to examine this population as available anti-mouse CD169 antibodies are not effective in paraffin-embedded tissues. We have tried these antibodies without success.

In bone, the importance of osteoblast apoptosis in homeostasis and pathologies is widely acknowledged [40], however, efferocytosis in this tissue remains under-investigated. When considering the three fates of osteoblasts (osteocytes, bone-lining cells, apoptosis), the greatest numbers undergo cell death (40–70%) [41]. While macrophages have been implicated in osteoblast efferocytosis [7–9], previous studies were limited to circumstantial evidence and/or utilization of in vitro techniques. Here, using the iCasp9 model, we provided direct in vivo evidence that while both osteoclasts and macrophages are capable efferocytes in bone, macrophages have a predominant role in apoptotic osteoblast clearance. This was not surprising given their well-recognized efferocytic functions across multiple systems [3] and their anatomical location within bone. Bone-associated macrophages, termed “osteomacs”, form canopy-like structures over osteoblasts [42] and thus, are appropriately positioned to facilitate their clearance. While osteomac-specific markers remain elusive, they have been characterized by CD169 expression [15]—a commonly used marker of efferocytic macrophages in spleen [39] and lymph node [43]. Rare efferocytic osteoclasts were detected herein but were predominantly positioned near the cartilaginous endplates where hypertrophic chondrocytes routinely undergo apoptosis [44]. We were unable to determine the nature of osteoclast efferocytic meal (i.e. chondrocytes versus osteocytes/osteoblasts) but the iCasp9 model could address this by crossing with chondrocyte-targeted Cre mice (e.g. Col10a1-Cre) [45].

In conclusion, our study presented the first mouse model of iCasp9 which achieves selective cell apoptosis and investigation into the concomitant efferocytosis. Aberrant efferocytosis is associated with numerous inflammatory diseases, autoimmune disorders and cancers [46–48]. Among pertinent examples, failure to clear apoptotic bodies drives lesion progression and plaque stability in atherosclerosis [49, 50] and in metastatic bone disease, macrophage efferocytosis of apoptotic cancer cells creates a microenvironment that promotes tumor progression [47, 48]. The iCasp9 mice can facilitate further investigation into these pathologies focused at elucidating the phenotypes of defective efferocytosis that will inform the development of corrective strategies. In addition, the iCasp9 mice could also be used as a cell-specific ablation strategy to investigate diseases driven by excessive cellular production (e.g. myeloproliferative disorders). The potential applications of this model are extensive given its unique feature of being controlled by any Cre-expressing mouse lines. While an impressive amount of knowledge has been gained on the dynamics of cell clearance, the heterogeneity of the process means that a mechanism learned from a particular cell/tissue type cannot be universally applied to others. The iCasp9 mice provide an in vivo cell/tissue-targeted approach that will surely bring forth novel insights into efferocytosis across different tissues, including how the process is regulated/dysregulated in homeostatic versus pathological conditions.

## Availability of data and materials

The raw and processed data that support the findings of this study is available at 10.6084/m9.figshare.21931131.v1.

## Supplementary information

Supplemental Material

Reproducibility Checklist doc
