# Supplementary material for: Caspase-9 driven murine model of selective cell apoptosis and efferocytosis
Source: Cell Death Dis. 2023 Jan 24;14(1):58. doi: 10.1038/s41419-023-05594-6 (PMC9873735; doi:10.1038/s41419-023-05594-6)
Supplement: Supplementary file 1 — Supplemental Material [file 41419_2023_5594_MOESM1_ESM.pdf]

SUPPLEMENTARY INFORMATION

Detailed generation of the iCasp9 mouse model

The generation of iCaspase9-T2A-eGFP transgenic mouse model involved three steps. The first step was to generate a genetically modified mouse line, designated R26-3XattP (R26P3), by knocking in three tandem attP sequences, namely landing pad, into mouse Rosa26 (R26) locus of the C57BL/6J genetic background. The second step integrated the iCaspase9-T2AeGFP transgene cassette flanked by attB sequences into R26P3 sites in the mouse genome by microinjecting the TARGATTTM transgene construct and ϕC31 integrase mRNA into the male pronucleus of heterozygous R26P3 zygotes. During embryonic development, ϕC31 integrase catalyzed site-specific DNA integration between transgene construct-contained attB sequences and the attP sequences borne by the embryonic genome, resulting in the iCaspase9-T2A-eGFP transgene cassette integration into the R26P3 locus in the genome. Post microinjection, embryos were transferred into CD1 pseudo-pregnant recipients to carry pups. The third step identified iCaspase9-T2A-eGFP transgenic F0 founders by PCR-based genotyping using a panel of PCR primers to confirm: (1) integration of iCaspase9-T2A-eGFP transgene with gene of interest primers (Supplemental Table 1), (2) attB and attP sequences exchange between the construct and the landing pad, and (3) site-specific insertion of the expression cassette of iCaspase9-T2A-eGFP or bacterial backbone to the landing pad through exchange of attB borne by the construct with attP in the landing pad. The second zygotic injection generated two clean site-specific F0 founders (out of 34 pups) which were then mated with C57BL/6J mice to generate F1 heterozygous pups (iCasp9$^{+/-}$). To confirm stable construct integration and subsequent progeny, PCR primers (Supplemental Table 1) were used to determine presence of iCasp9 construct and employed for maintenance genotyping. The objective of the knock-in generation was to obtain the conditional expression model, using a Lox-STOP-Lox-iCaspase9-IRES-eGFP expression cassette. The iCasp9$^{+/-}$ mice were bred to obtain homozygous iCasp9$^{+/+}$ mice. When crossed with

specific Cre driver mouse lines, the Cre recombinase will removed the stop sequence and both iCaspase9 and EGFP proteins will be translated at equimolar quantities from the same bicistronic mRNA containing the T2A sequence (1).

Reference

1. Trichas G, Begbie J, Srinivas S. Use of the viral 2A peptide for bicistronic expression in transgenic mice. BMC Biol. 2008;6(1):40.

Tables

Supplemental Table 1. Primers used for genotyping.

iCasp9	Forward Primer	Reverse Primer	bp
iCasp9 Gene of Interest	iCasp9: CGCCATATCTAGTTTGCCCACACC	EGFP: CTGAACTTGTGGCCGTTTACGTC	448
Wild type (no iCasp9)	CACTTGCTCTCCCAAAGTCGCTC	CGAGGCGGATCACAAGCAATAATAACC	449
CD19-Cre	GCGGTCTGGCAGTAAAAACTATC	GTGAAACAGCATTGCTGTCACTT	100
CD19-Cre (WT)	CCTCTCCCTGTCTCCTTCCT	TGGTCTGAGACATTACAATCA	477
OCN-Cre	TCCAATTTACTGACCGTACACCAA	CCTGATCCTGGCAATTTCGGCTA	545

Supplemental Table 2. Immunofluorescence and immunohistochemistry antibodies.

Antibody	Source	Identifier	Dilution	Incubation time	Antigen retrieval
EGFP	Abcam	ab13970	1:500	2h	Proteinase K (50µg/ml)
CD19	Abcam	ab245235	1:1000	2h	Tris-HCl (pH 9.0)
Cleaved caspase 3	Cell Signaling	9664	1:1000	Overnight	Tris-HCl (pH 9.0)
Osteocalcin	Thermo Fisher	PA578870	1:200	1.5h	Proteinase K (50µg/ml)
Ly6G	Abcam	ab238132	1:1000	Overnight	Proteinase K (50µg/ml)
F4/80	Abcam	ab6640	1:200	1.5h	Proteinase K (50µg/ml)
CD11c	HistoSure	HS-375 003	1:100	1.5h	Sodium citrate (pH 6.0)

Figures

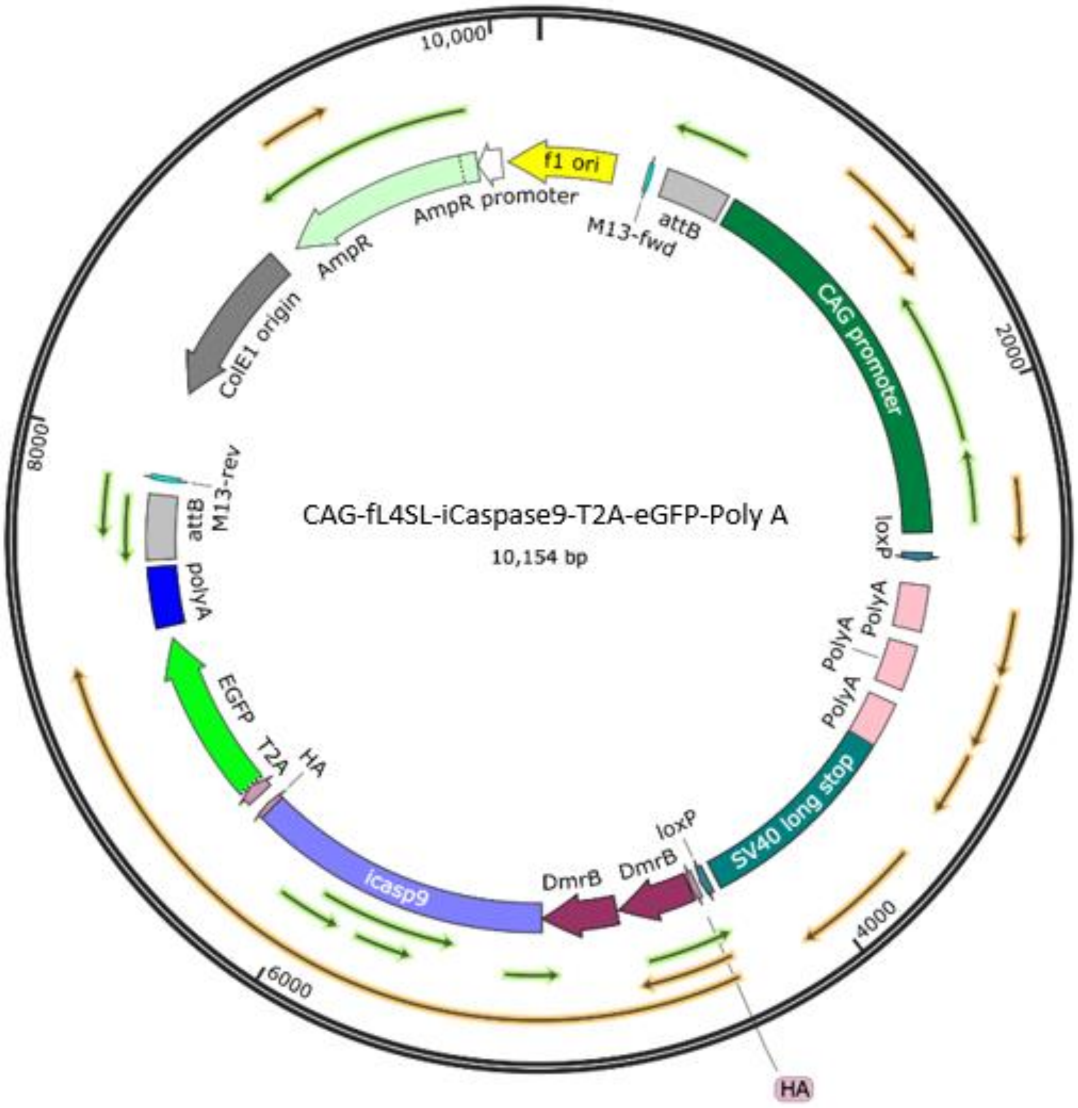

Figure S1. iCaspase9-EGFP transgenic mouse model plasmid generation. In collaboration with Applied Stemcell (ASC), the construct CAG-fL4SL-iCaspase9-T2A-eGFP-Poly A was made by inserting a synthetic fragment containing a leading Kozak box before the start codon, HA tag-F36V/F36V mutant fused with Caspase9 cDNAHA tag, followed by T2A-eGFP with a stop codon into a TARGATT™ vector pTARGATT6.1 (proprietary property of ASC). The expression cassette is driven by CAG promoter in the construct. The intact expression cassette CAG-fL4SL-iCaspase9-T2A-eGFP-Poly A is flanked by two attB sites. The correct integration of the TARGATT construct is shown.

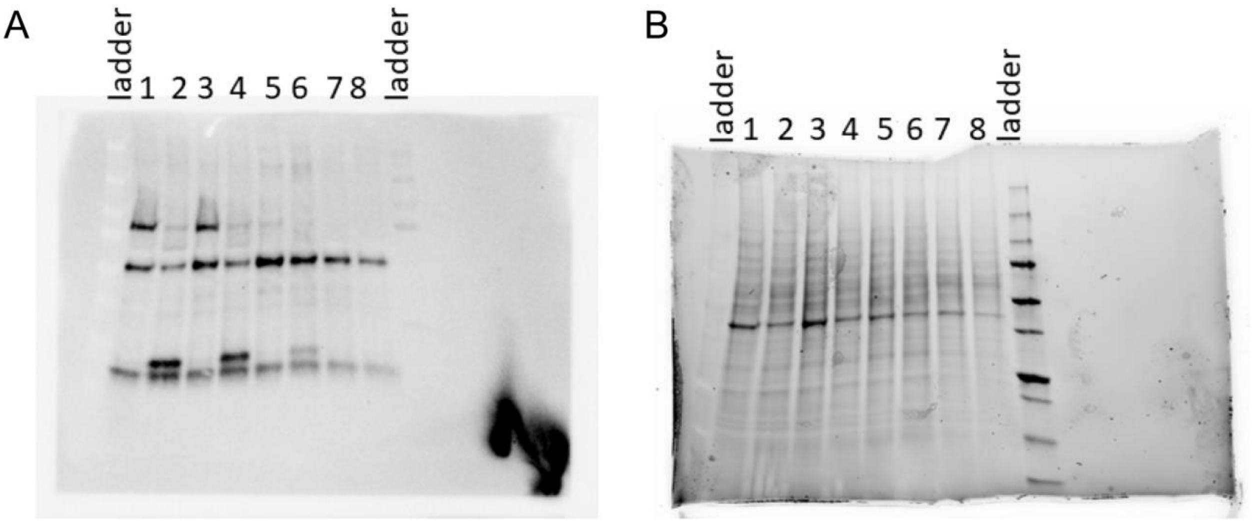

Figure S2. Uncropped Western Blot (A) and protein gel (B) corresponding to Figure 1C.

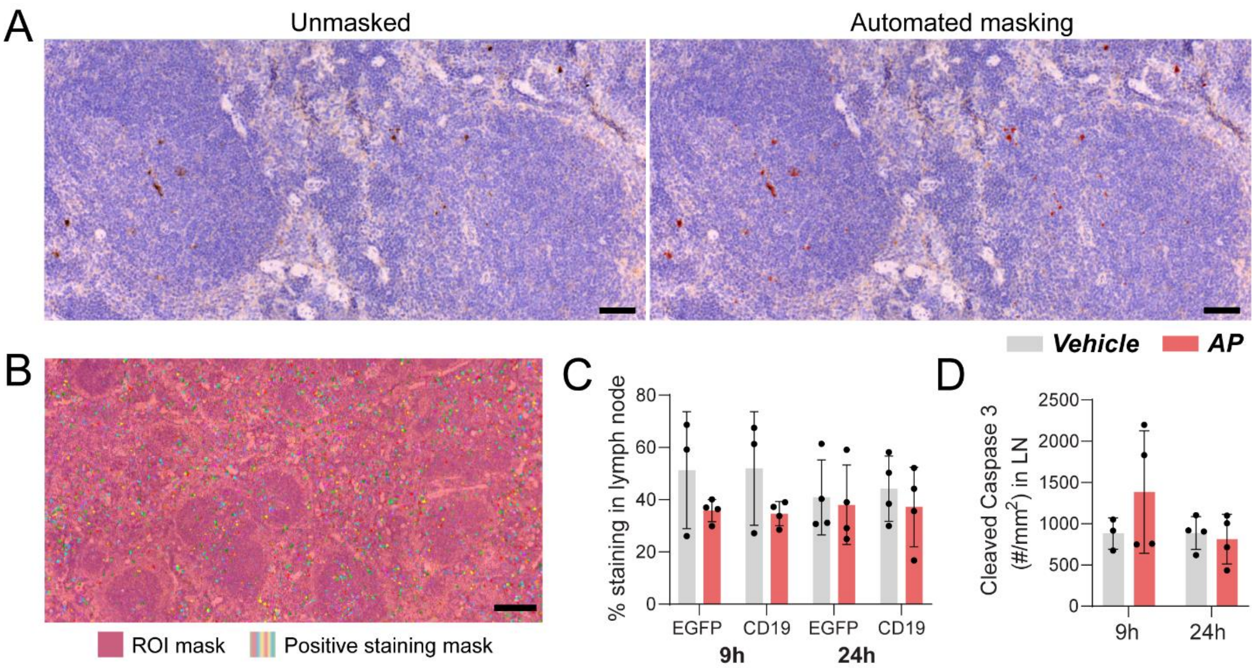

Figure S3. Impacts of AP treatment in different tissues of CD19-Cre$^{+/-}$iCasp9$^{+/+}$ mice. (**A**) Unmasked and masked versions of the same area of the spleen. Masked image shows precise detection of DAB staining (red) by the algorithm developed in AIVIA software. (**B**) Example of a magnified output image from the AIVIA software showing masking of the region of interest (ROI) and DAB staining. Quantification of (**C**) percent EGFP^{+} and CD19^{+} staining, and (**D**) cleaved caspase-3 number in serial inguinal lymph node sections. Statistical significance was determined using two-way ANOVA with Sidak's multiple comparisons test. Error bars represent standard deviation. n=3-4/group. Scale bar: 50µm (A), 500µm (B).

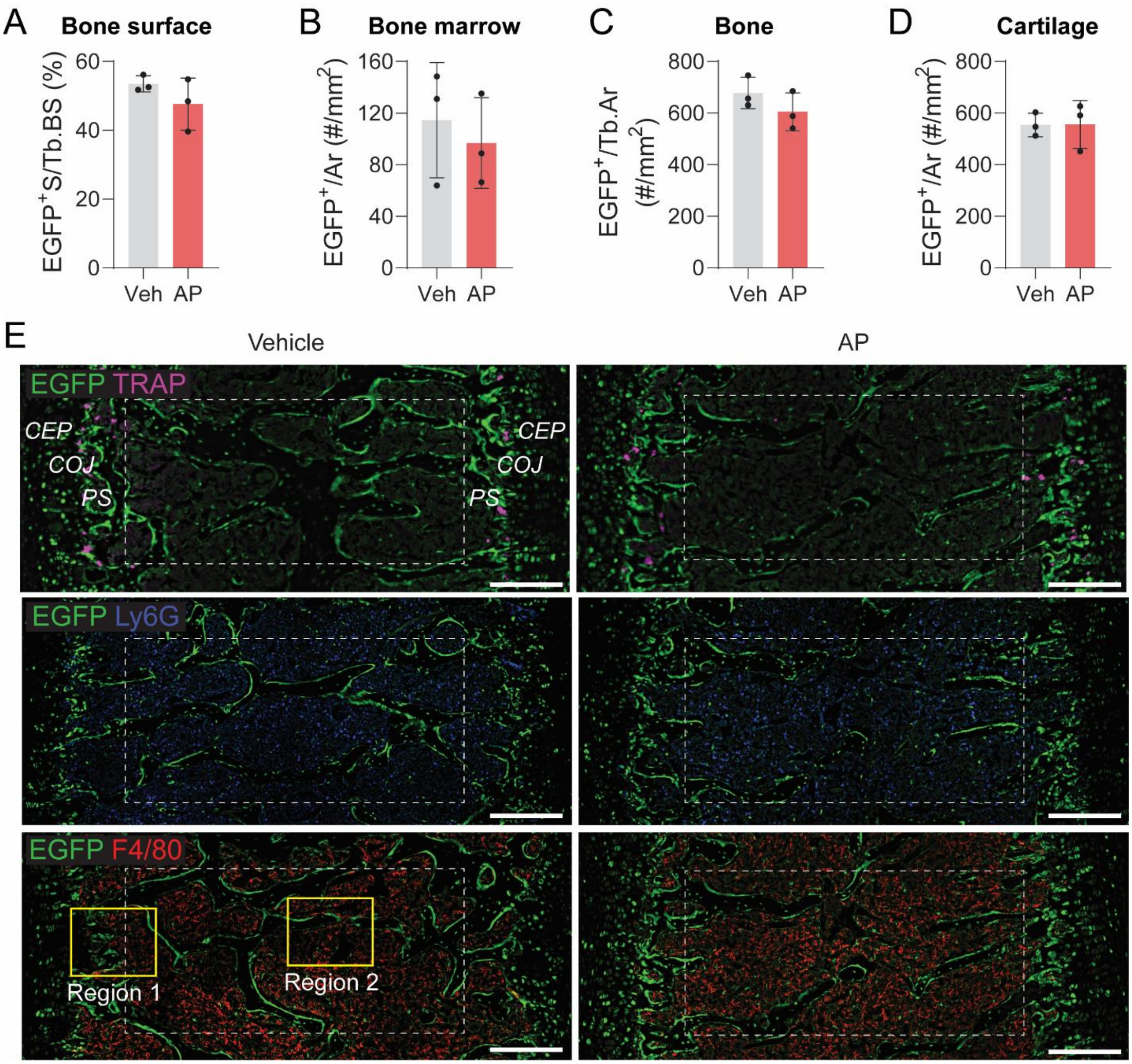

Figure S4. Impacts of AP treatment in OCN-Cre$^{+/-}$iCasp9$^{+/+}$ mice. Quantification of (**A**) EGFP^{+} surface (S) per trabecular bone surface (Tb.BS), (**B**) number of EGFP^{+} signal in the bone marrow per tissue area (Ar), (**C**) EGFP^{+} osteocytes expressed as number per trabecular bone area (Tb.Ar) and (**D**) number of EGFP^{+} signal per area (Ar) of cartilaginous endplates at 9h post treatment with vehicle (Veh) or AP. (**E**) Distribution of EGFP staining with TRAP (magenta), Ly6G (blue) or F4/80 (red) in the whole vertebra at 24h post treatment with vehicle or AP. Sections were from the same vehicle- or AP-treated animal but were taken at different sectional depths. CEP – cartilaginous endplate, COJ – chondro-osseous junction, PS – primary spongiosa. Dotted borders mark the

region consisting predominantly of vertebral marrow and trabecular bone. Yellow boxed areas indicate the positioning of the regions imaged in Figure 5A. Region 1 was taken in the COJ and Region 2 was taken at the middle of the vertebra. Statistical significance was determined using two-tailed unpaired t-test. Error bars represent standard deviation. n=3/group. Scale bar: 200μm (E).
